# Complexity in the dengue spreading: A network analysis approach

**DOI:** 10.1371/journal.pone.0289690

**Published:** 2023-08-07

**Authors:** L. L. Lima, A. P. F. Atman

**Affiliations:** 1 Programa de Pos-Graduação em Modelagem Matemática e Computacional, Centro Federal de Educação Tecnológica de Minas Gerais, Belo Horizonte, Minas Gerais, Brazil; 2 Departamento de Física, Centro Federal de Educação Tecnológica de Minas Gerais- CEFET-MG, Belo Horizonte, Minas Gerais, Brazil; 3 National Institute of Science and Technology for Complex Systems-CEFET-MG, Belo Horizonte, Minas Gerais, Brazil; Instituto Nacional de Salud Pública: Instituto Nacional de Salud Publica, MEXICO

## Abstract

In an increasingly interconnected society, preventing epidemics has become a major challenge. Numerous infectious diseases spread between individuals by a vector, creating bipartite networks of infection with the characteristics of complex networks. In the case of dengue, a mosquito-borne disease, these infection networks include a vector—the *Aedes aegypti* mosquito—which has expanded its endemic area due to climate change. In this scenario, innovative approaches are essential to help public agents in the fight against the disease. Using an agent-based model, we investigated the network morphology of a dengue endemic region considering four different serotypes and a small population. The degree, betweenness, and closeness distributions are evaluated for the bipartite networks, considering the interactions up to the second order for each serotype. We observed scale-free features and heavy tails in the degree distribution and betweenness and quantified the decay of the degree distribution with a *q*–Gaussian fit function. The simulation results indicate that the spread of dengue is primarily driven by human-to-human and human-to-mosquito interaction, reinforcing the importance of controlling the vector to prevent episodes of epidemic outbreaks.

## Introduction

Complex networks approach has been widely applied in many fields of science [1, 2]. The structure of a network can be found in different phenomena, from the spread of diseases to the world wide web [3]. The spread of diseases in complex networks emphasizes the role of network topology in the study of epidemiological modeling [4]. For instance, the complex networks approach was one of the tools used to study the recent COVID-19 pandemic [5, 6]. However, this approach was used many times before to study the spreading of diseases such as HIV [7], influenza [8–10], Ebola [9], Zika [9, 11, 12], chikungunya [11, 12] and dengue [11–13].

Heterogeneity in the population and environment often plays a crucial role in determining the occurrence of an epidemic [14]. It is estimated that about 20% of the infected by a transmissible disease are responsible for 80% of infections (20/80 rule) -including vector-borne parasites and sexually transmitted pathogens [15]. We recently investigated the emergence of superspreaders in dengue and COVID-19 infection networks [16, 17]. Observing different transmission networks between vectors and humans up to the second generation, we show that, despite the human-to-human transmission network following the 20/80 rule, the other networks (human-to-mosquito, mosquito-to-mosquito, and mosquito-to-human) did not follow this rule [16].

Dengue is a viral disease transmitted by female mosquitoes whose main vector in urban areas is *Aedes aegypti* [18]. This disease is currently considered the most prevalent, widely distributed, and fastest-spreading mosquito-borne viral disease globally [19, 20]. According to the World Health Organization, in March 2023, approximately half of the world’s population is at risk of dengue (an estimated 100 to 400 million infections annually) [21].

The spread of dengue is related to factors such as climate change [22], population immunity, environmental characteristics [23], mobility [24], and how the disease spreads in a network. In Singapore, a study showed that the dengue epidemic from 2000 to 2005 organized itself into a scale-free transmission network as the outbreak progressed [25]. Another study using networks identified that the incidence of dengue in Selangor, Malaysia, follows a scale-free behavior [26].

Dengue has established itself in endemic and epidemic transmission cycles around the world [27]. Until World War II, dengue epidemics occurred every 10 to 30 years [28]. Several areas were favorable for *Ae. aegypti* in North and South America in the late 20th century [29]. One or more serotypes can be endemic in the same population, causing outbreaks every 3 to 5 years [30]. Currently, four serotypes of the disease are in circulation worldwide, and a fifth serotype was isolated in Malaysia in 2015 from samples of a human infected in 2007 [31].

One of the reasons it is difficult to obtain actual transmission data to build a dengue-infected network is that it is a mosquito-borne vector disease, and while it is feasible to track humans, it is almost impossible to identify which mosquitoes have infected humans. To overcome this problem, we developed an agent-based model that simulates dengue transmission dynamics in an urban area [16]. This model allows tracking of disease transmission and subsequent analysis of the infection network.

## Materials and methods

Unlike other approaches that consider different network structures *a priori* [32], our model analyses the self-organized network that emerges from the agents’ behavior. A comparison of the model and field experiments [33] was performed previously to guarantee the validity of the results. In this work, we study the bipartite networks formed by the dengue infection spreading and characterize the main network measures up to the second-order interaction. The degree, betweenness, and closeness distribution are evaluated and discussed. The degree distribution presents scale-free features, and we performed a successful fitting procedure with a *q*–Gaussian function, confirming the heavy tails of the distribution. The multiplex network also was built considering each serotype as a layer.

### Agent-based model

Mosquitoes and humans are agents and can assume a finite number of states updated synchronously at each time step (Table 1). However, only female mosquitoes were considered in the simulation, as they are the ones that transmit the disease to humans [34, 35]. This is the same model used by Lima and Atman [16].

**Table 1:** Agents’ status and description.

State	Mosquito	Human	Definition
**Susceptible**	Yes	Yes	Agent has not yet been infected and can become infected.
**Incubated**	Yes	Yes	Agent is infected and contracts the virus (one or more serotypes). However, it still not transmitting the disease to other agents.
**Infected**	Yes	Yes	Agent is infected and can transmit the disease to others.
**Recovered**	No*	Yes	Agent has recovered from the serotype and remains immune for this serotype(s).

*Mosquitoes cannot be recovered because once infected, they remain infected for the rest of its life, even after repeated meals of human blood [34].

The model was calibrated using empirical data [33, 36–38] from mosquito traps. To reproduce the results, we installed traps in the model and simulated the capture of mosquitoes to compare with the empirical data. The calibration showed the best results with populations of 500 humans and 108 mosquitoes per block [16]. However, despite calibrating the model including the mosquito traps, these traps were not used in the model for the analysis presented in this paper since the aim is to build infection networks only.

**Fig 1:**
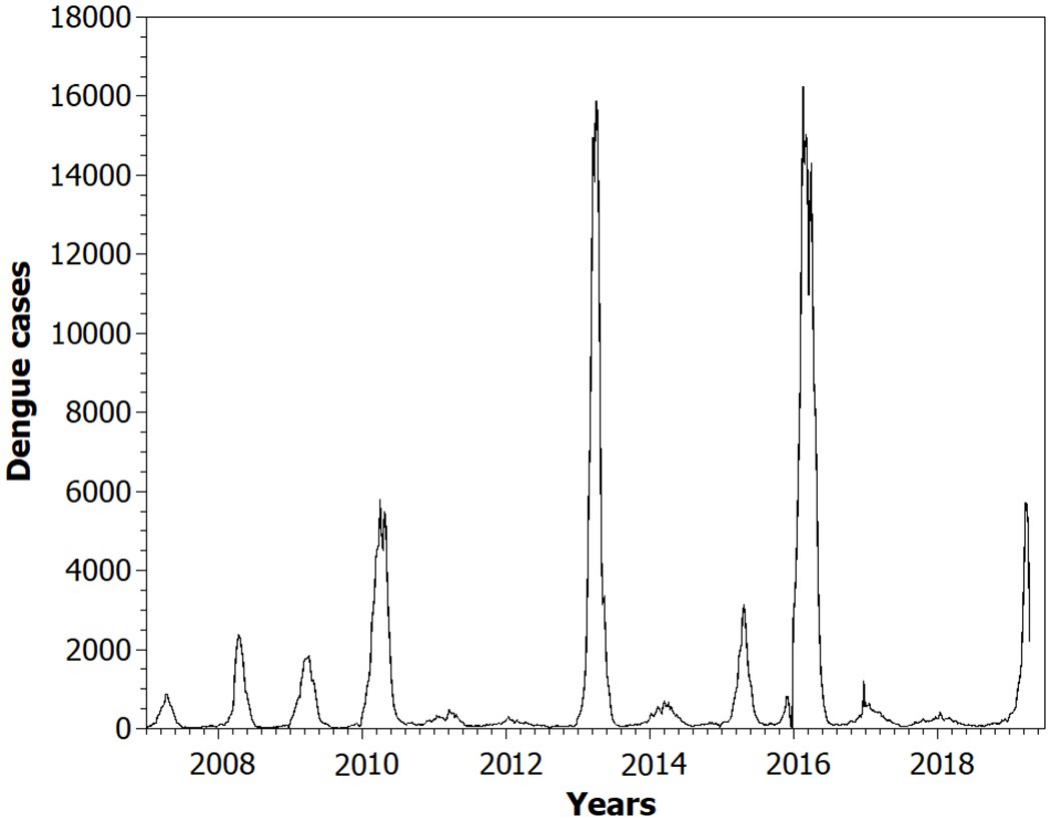
Dengue cases in Belo Horizonte from 2007 to 2019.

We considered local precipitation and temperature as attributes of the environment in the model. We used the average daily temperature and precipitation data from 2000 to 2016 from the Pampulha Meteorological Station in Belo Horizonte, Minas Gerais, Brazil [16]. This is the same city where mosquito trap data were collected [33]. If it rained and if the maximum temperature was less than 30°C and the minimum temperature was greater than 20°C we increased the site attribute by 50%. These are ideal conditions for eggs to hatch [39]. The number of dengue cases from 2007 to 2019 is shown in Fig 1. It is worth noting that the years we simulated are non-specific years. However, we considered the 16-year average of precipitation and temperature data to get better data on the city’s climate in the simulation. The meteorological data are a daily average of the value for these 16 years varying by the standard deviation (so the two simulation years are not the same).

Simulation time was two years, and each time step equals one minute. Humans move every hour, while mosquitoes fly every minute. Both perform a random walk and move with Moore’s neighborhood (they can stay where they are or go to one of the eight neighbors in a square lattice) [16]. However, the mosquito’s random walk is biased by a quenched noise weighted by the environment fitness around it. It was possible to assign each square meter to an average of pixels in the image using remote sensing and a satellite image of the simulated urban area (a residential block). This quantity is associated with one possible pavement, and a fitness value is attributed to the site. The mosquito then prefers to fly to areas with water and vegetation in the model (where it can reproduce) instead of to areas with paving.

The dynamics of the model happen with humans and mosquitoes interacting in the matrix of the environment. Mosquitoes can perform three actions [16]: fly (to another location or the same location), reproduce (if the location is suitable for oviposition and bite at least one human), and try to bite a human. If a human and a mosquito are in the same location, the mosquito will choose to bite the human with probability 80%. Otherwise, the mosquito will choose one of the two remaining options with equal probability.

The model starts with the entire susceptible population and infects five mosquitoes in the first time step. If a mosquito is infectious and bites a human, it transmits all the serotypes it has to the human except the ones the human is immune to it. The same happens if the mosquito bites a human and the human is in an infectious state; that is, the mosquito is infected with all the serotypes that the human is transmitting, except for those it has already been infected with it. Mosquitoes and humans can transmit more than one serotype simultaneously if infected with more than one serotype [40, 41].

Four dengue serotypes (DENV-1, DENV-2, DENV-3, and DENV-4) were considered in the simulation. DENV-1 was inserted at the beginning of the simulation, and the other serotypes were in a sequence with a 6-month interval between each one (DENV-2, DENV-3, and DENV-4, respectively—the same order as they were registered in the simulated city). Humans have matrices to record their infectious status. After a position in the immunity matrix is filled, it can no longer be reset to zero so that the human cannot be infected again by that serotype [16].

The model output data has been averaged over 100 runs but with the same interval for inserting a new serotype. Then we were able to build a disease transmission network between mosquitoes and humans [16], and we have observed that although human-human networks have superspreaders agents and obey the 20/80 rule, this does not happen in the others. It may be a characteristic of networks involving vectors and hosts [16].

### Networks

Both mosquitoes and humans are nodes in the network. Networks were built connecting mosquitoes and humans according to who transmits the disease to whom (direct network) [16]. Each infected agent is a network node, and not-infected agents were not considered in the networks.

We built four types of networks (the same as Lima and Atman [16]): human-to-human (not considering mosquitoes between these transmissions), mosquito-to-mosquito (not considering humans between these transmissions), human-to-mosquito, and mosquito-to-human. It is worth noting that the analyzed model’s results are the same for all four networks. The differences occurred in the construction of networks. For example, we remove the mosquito from the network (intermediate node) to build the human-to-human network. For the mosquito-to-mosquito network, we remove the human node.

We then calculated three measures to analyze and compare: degree, betweenness centrality, and closeness centrality. The degree of a node *v* is the number of links connected to *v*. We consider only the out-degree since the networks are from an infectious disease spreading only once for each agent.

The betweenness centrality of a node *v*, *B*(*v*), is given by Eq 1:

$$\begin{array}{c} B\left(v\right)=\sum_{s\neq v\neq t}\frac{\sigma_{st}\left(v\right)}{\sigma_{st}}\;, \end{array}$$ (1)

where *σ*_*st*_ is the total number of shortest paths from node *s* to the node *t* and *σ*_*st*_(*v*) is the number of these paths that pass through *v*. We normalized it by dividing *B*(*v*) by (*N* − 1)(*N* − 2) since the graph is directed. *N* is the number of nodes in the network.

The closeness centrality is given by *C*(*v*) in Eq 2.

$$\begin{array}{c} C\left(v\right)=\frac{N-1}{\sum_{u}d\left(v,u\right)}\;, \end{array}$$ (2)

where *d*(*v*, *u*) is the distance (number of nodes) in the shortest path between vertices *v* and *u*. Eq 2 is normalized due to the factor (*N* − 1) in the numerator.

The data were fit using a q-Gaussian distribution [42]. The *q*–Gaussian distribution was introduced by Tsallis to describe systems subjected to long-ranged correlations in the context of non-extensive thermodynamics [42, 43]. The fitting function is given by Eq 3,

$$\begin{array}{c} f\left(x\right)=\frac{\sqrt{\beta }}{C_{q}}e_{q}\left(-\beta x^{2}\right)\;, \end{array}$$ (3)

where *β* and *C*_*q*_ are treated as adjusting parameters, and the *q*−exponential is given by:

$$\begin{array}{c} e_{q}\left(x\right)={\left[1+\left(1-q\right)x\right]}^{\frac{1}{1-q}}\;. \end{array}$$

For *q* = 1, we recover the usual Gaussian distribution. *q* > 1 leads to platykurtic distributions while *q* < 1 to leptokurtic ones.

## Results

**Fig 2:**
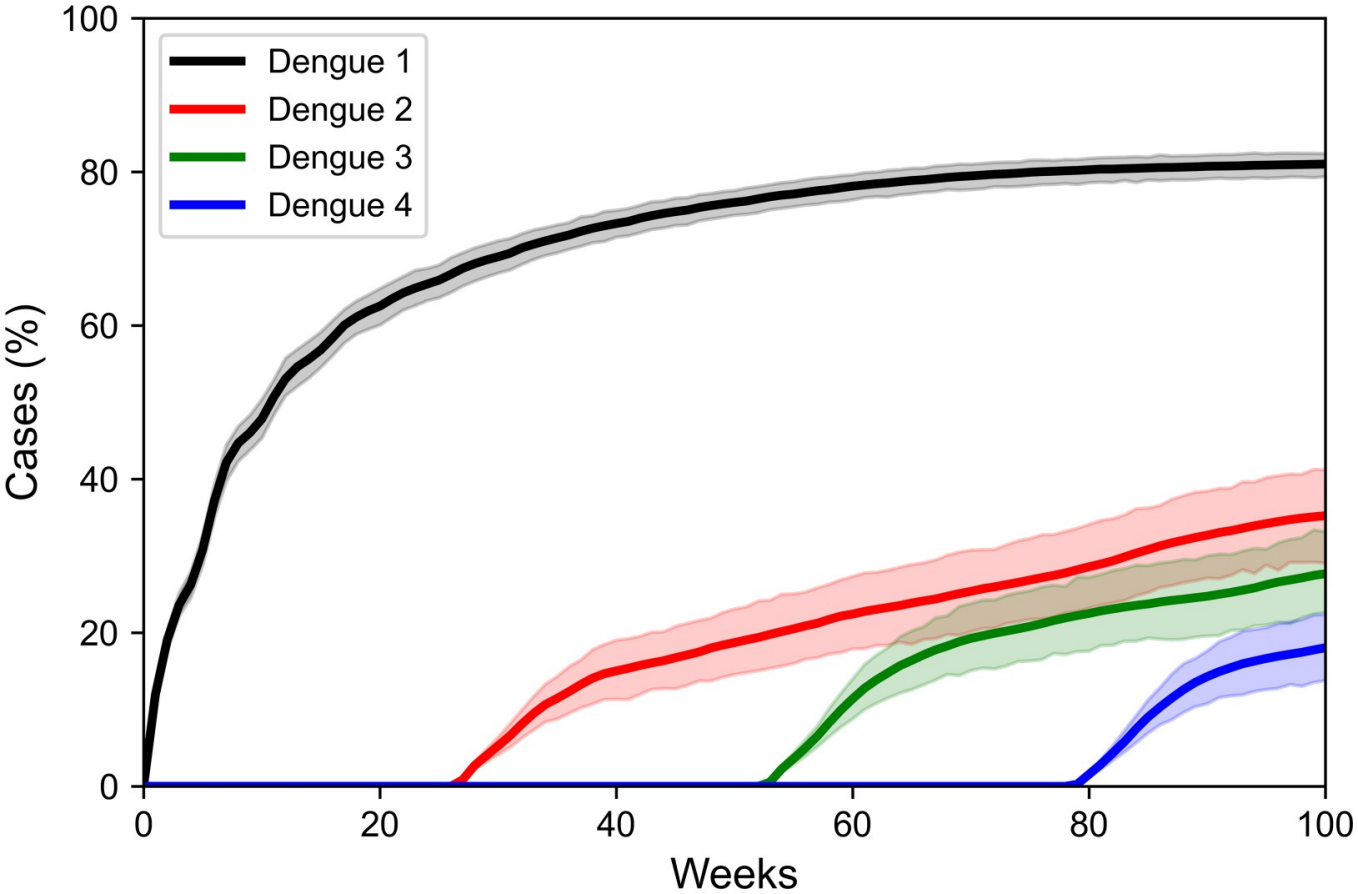
Cases of dengue during 2-year simulation. Blue lines represent the mean (along with 95% confidence intervals in shaded blue) across 100 runs.

Dengue cases accumulated over time are shown in Fig 2. It shows that a large number of humans (about 60% of the population) were infected with the disease over the simulation period for dengue 1. About 35% were infected with dengue 2, 30% with dengue 3, and 20% with dengue 4. The earlier a serotype was inserted into the model, the greater the number of people infected by that serotype. Our tests showed that there is not much difference related to the mean and standard deviation of the number of infected for more than 50 runs, so we chose to use 100 runs as an optimal number in this study.

**Fig 3:**
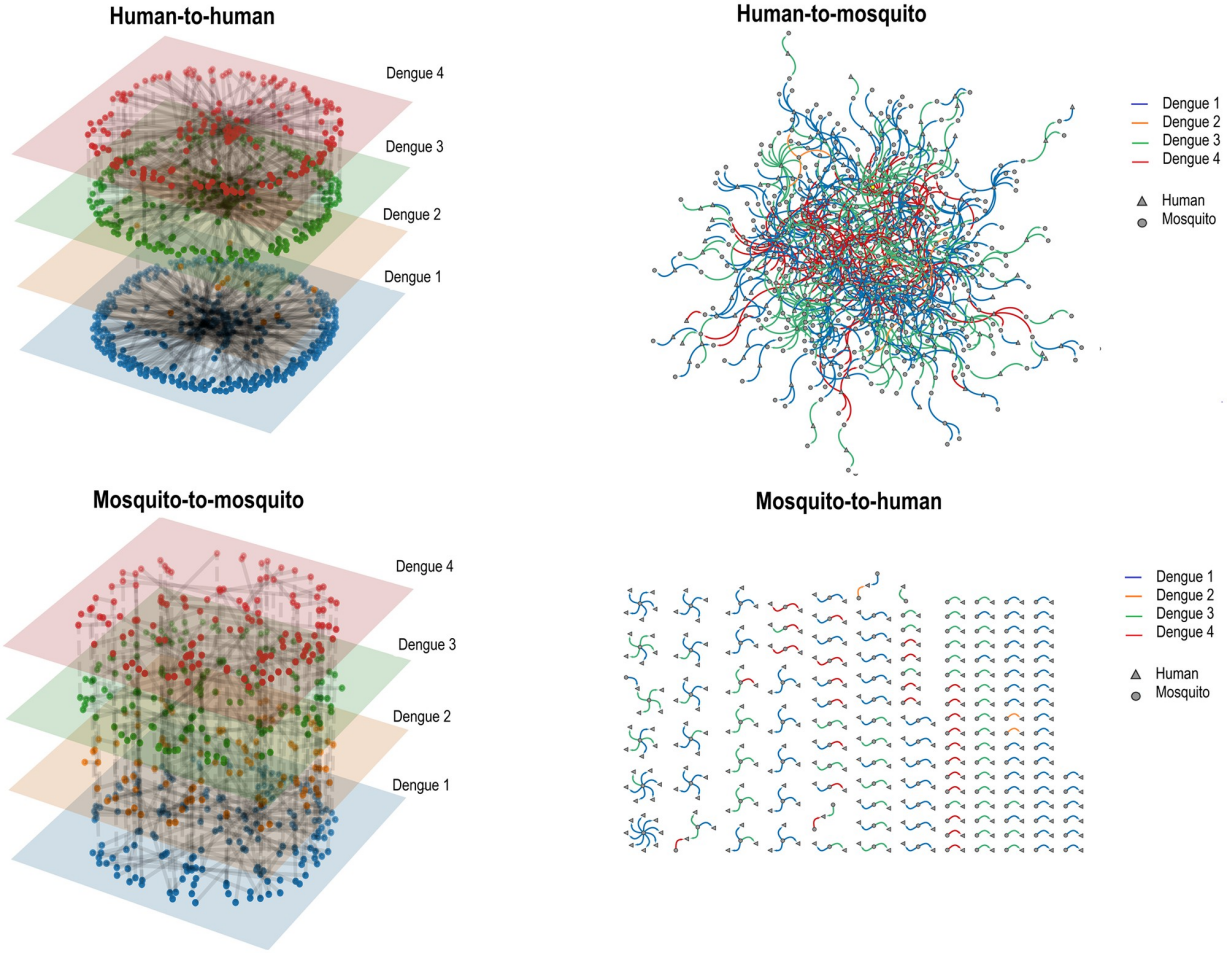
Networks built from the model result (1 run). Colors represent different serotypes of dengue.

Fig 3 shows an example of the model’s infection networks. The human-to-human and mosquito-to-mosquito networks were built by serotypes and layers. On the other hand, the human-to-mosquito and mosquito-to-mosquito bipartite networks were differentiated by serotypes by edge colors.

**Fig 4:**
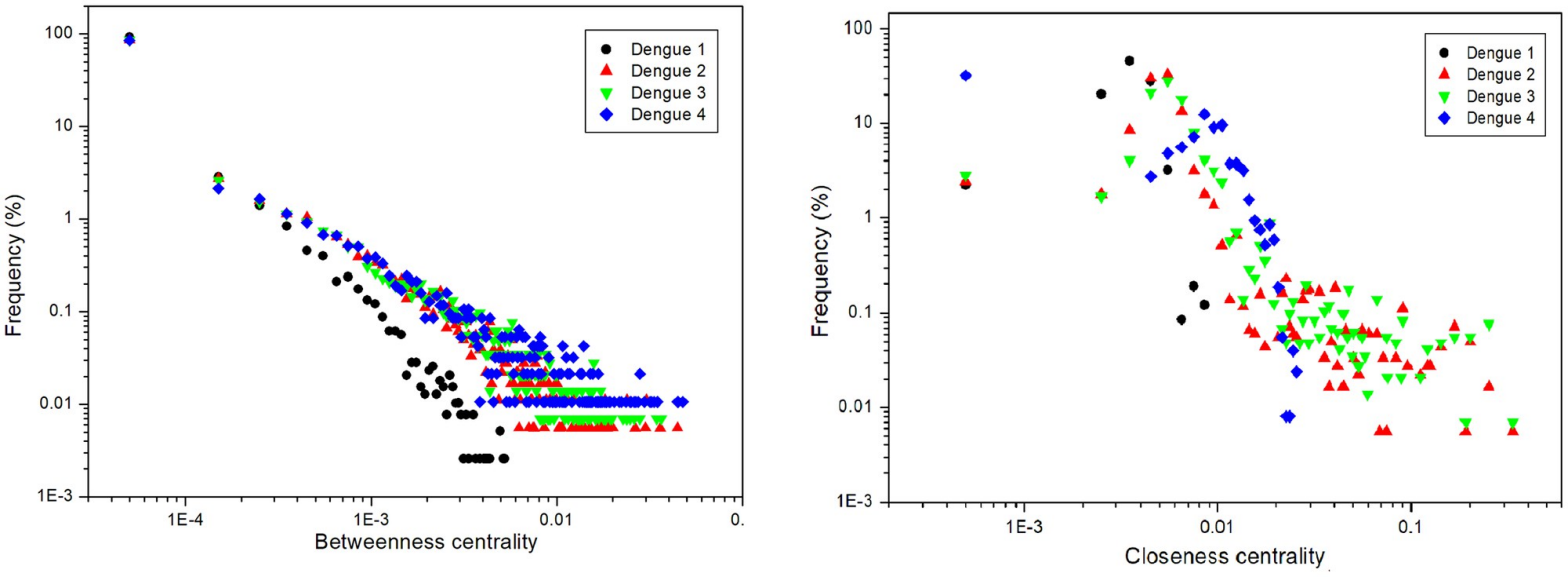
Human-to-human network results. Betweenness centrality and closeness centrality. Data from 100 runs.

**Fig 5:**
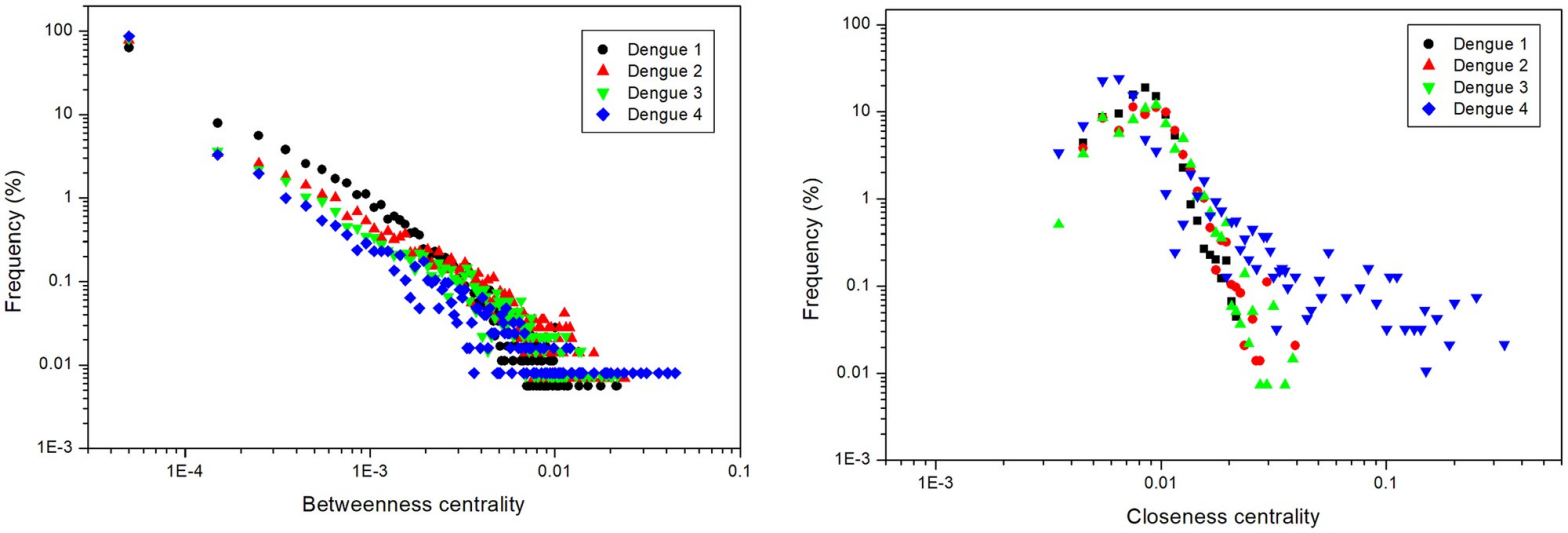
Mosquito-to-mosquito network results. Betweenness centrality and closeness centrality. Data from 100 runs.

Fig 4 shows the distribution of betweenness centrality and closeness centrality of human-to-human infection networks, and Fig 5 of mosquito-to-mosquito networks. Most nodes in human-to-human and mosquito-to-mosquito networks have low betweenness values; i.e., these nodes are not essential for disease transmission along the network. On the other hand, nodes with higher betweenness are important to keep the transmission active. As for closeness, while most nodes also do not have high closeness values, most other nodes have intermediate values, indicating that these nodes are closer to the rest of the network and essential for transmission. It is worth noting that the degree is slightly higher, and the betweenness and closeness centralities are slightly lower for DENV-1 because this serotype was the first to be inserted into the model, having more time to spread than the others. Closeness centrality also behaves slightly differently for DENV-4 from the others because it was the last serotype to be inserted in the simulation.

We observed power-law distributions to betweenness centrality, indicating the presence of superspreaders in the networks. As observed previously for degree distribution [32], scale-free distributions are essential in epidemic infections, and it is striking that we observe this feature in the self-organized networks emerging from the model.

**Fig 6:**
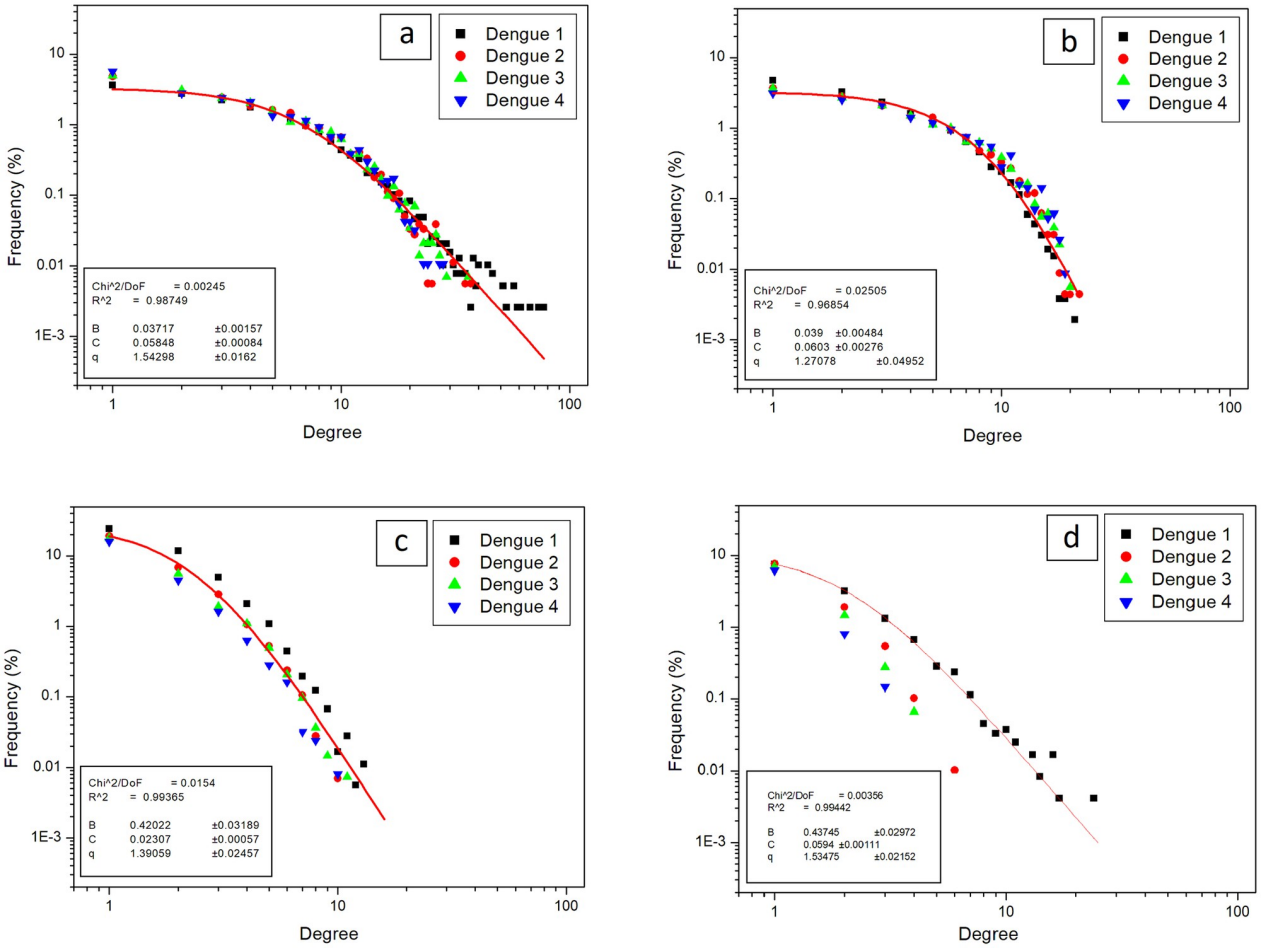
Degree of networks. Lines represent the fit using q-Gaussian distribution—Eq 3. a: Human-to-human. b: Human-to-mosquito. c: Mosquito-to-human 3. d: Mosquito-to-mosquito.

Fig 6 shows the degree distribution of human-to-human, human-to-mosquito, mosquito-to-human and mosquito-to-mosquito networks. Since it has a single parameter *q* to characterize the deviation from Gaussian distribution, it was intuitive to interpret the fit results with this function. As shown in Fig 6, the matching of the fitting function to the data was remarkable. Despite some networks having a little poor data, the distribution was able to reproduce the functional behavior of the degree distribution. The most robust result was obtained for the human-to-human network, with a *q* = 1.54. This value of *q* can be interpreted as a long-range correlation in spreading the disease among humans. It is compatible with the model since humans displace more than mosquitoes and have a longer life cycle.

Table 2 shows the agents responsible for infections in each network and the percentages of human and mosquito populations infected by each serotype.

**Table 2:** Percentage of infective and infected. Results of 100 simulations.

Serotype	H-H	H-M	M-M	M-H	Humans infected	Mosquitoes infected
**DENV-1**	11.54%	2.49%	2.01%	1.78%	66.12%	13.33%
**DENV-2**	11.26%	2.48%	1.99%	1.74%	64.35%	13.58%
**DENV-3**	11.32%	9.08%	2.01%	1.74%	65.29%	12.96%
**DENV-4**	10.92%	8.97%	1.97%	1.68%	63.88%	12.73%

H-H: human-to-human; H-M: human-to-mosquito; M-M: mosquito-to-mosquito; M-H: mosquito-to-human.

The degree calculation made it possible to estimate the percentage of infective and infected in the system. In human-to-human networks, between 10.92% to 11.54% of humans infected are indirectly responsible for human infections. Equally, between 2.49% and 9.08% of humans are responsible for all mosquito infections (12.73% to 13.58% of the mosquito population) in human-to-mosquito networks. In mosquito-to-mosquito networks, between 1.97% to 2.01% of infected mosquitoes indirectly infect other mosquitoes. On the other hand, between 1.67% to 1.78% of the mosquito population infected humans (63.88% to 66.12% of the human population).

## Discussion

Dengue is a cyclical disease that presents recurrent epidemic outbreaks, with different serotypes circulating simultaneously [30]. Its behavior has a non-linear relationship with climatic conditions, population immunity, the built environment, and human mobility [23]. All these factors contribute to the difficulty of predicting and reproducing epidemic outbreaks at the simulation level. However, our model can reproduce the disease’s dynamics in small populations. As the model is a closed system with a small population (500 humans) that is totally susceptible and limited to a small space (one block), the disease spreads quickly, reaching about 60% of the population in two years of simulation (Fig 2).

The human-to-human network presents a degree distribution that shows that a few humans are indirectly responsible for many infections of other humans (with mosquitoes as intermediaries in transmission) (Fig 6). This means that only a tiny portion of those infected humans are responsible for most infections, as shown by studies of superspreaders [15]. The values of infective and infected in Table 2 confirm that only a small portion of those infected are responsible for infections in the system. On the other hand, mosquito-to-mosquito networks are the ones with the lowest degree (Fig 6). This can be explained because the mosquito population has a shorter lifetime (mosquitoes live approximately 45 days in the model, while the human population is renewed by 5% each year). Consequently, time is insufficient for one mosquito to have a high degree, that is, to transmit to many humans.

Many agents have a betweenness centrality value close to zero (Figs 4 and 5). However, there are mosquitoes and humans with a high betweenness centrality value, which may indicate that the infection routes are centered on certain agents that connect several other agents. This may indicate that an effective measure to reduce the number of cases in the real world is to reduce the mosquito population and prevent infected humans from being bitten, thus breaking networks and eliminating disease transmission routes. For closeness centrality, some humans have higher values than others (Fig 4), indicating that they are closer to the rest of the network, while mosquitoes have values that are not so high (Fig 5). This reinforces the idea of preventing infected humans from being bitten by mosquitoes. The same measures used by healthy humans to avoid mosquito bites can be used: repellent, mosquito netting, and others.

While the human-to-human and mosquito-to-mosquito networks represent indirect transmission, as they need a mosquito and a human as intermediaries, respectively (the model did not consider transmission from female mosquitoes to eggs), the human-to-mosquito and mosquito-to-human networks are bipartite. Being able to be modeled as bipartite networks is a characteristic of vector-borne diseases [44].

Dengue transmission showed the behavior of a scale-free network in the model (Fig 6). This behavior has already been found in the literature during an outbreak of the disease in Singapore, in which study the authors used the geographical network of clusters of cases. [25]. In vector-borne diseases, in which transmission can be described in bipartite networks, extreme aggregation of vectors in hosts can influence epidemic threshold behavior [32]. Infections can spread through scale-free networks regardless of these diseases’ spread rates [4, 26].

The q-Gaussian distribution generalizes the Gaussian distribution allowing a third parameter—the *q* index, to control the decaying of the distribution tails. For *q* < 1, the distribution decays faster than Gaussian, and for *q* > 1, it decays slower. Thus, a value of *q* greater than one indicates that the distribution of larger values is more frequent than the expected normal distribution. A remarkable result lying the *q* and the diffusion exponent *α* is that *α* = 2/(3 − *q*), which implies that for *q* = 1, we have a normal diffusion, and for *q* larger than one, we have a super-diffusion regime, with *α* > 1 [42]. In the case of the human-to-human network, degree distribution indicates that the presence of superspreaders, that is, individuals with large degree values, is more frequent. These superspreaders infect more individuals, making the infection more extensive since each individual infected will spread the virus along their path.

Although the network results are promising, this model has limitations. The first is that it is a closed system with small populations of mosquitoes and humans that may represent small urban areas but not the full complex urban dynamics of the disease. The model was also simulated for a small time interval, covering only one dengue cycle of each serotype. A more extensive temporal and spatial analysis would be interesting to evaluate changes in the networks studied in this work.

Even though its limitations, this model can be used as a starting point for a more extensive and in-depth analysis. Unlike an airborne or sexually transmitted disease, it is not feasible to measure the transmissibility of each mosquito. Then this model can help understand how the disease spreads in different scenarios.

Computational modeling aims to improve the acknowledgment of the underlay dynamics of systems that are complex to understand. It is the case of dengue, in which the serotype dynamics still need to be fully understood. By studying the microscopic transmission of the disease through the infection graph, we showed some features, such as superspreading, which can help improve public policies to be more effective in controlling the spreading. The model can be adapted for other vector-borne diseases in which it is possible to measure the number of infected humans but not the number of vectors. The results found in this work reinforce the idea that complex networks can be used to study the spread of infectious diseases, contributing to decision-making in public health.
